# Observation of superconducting vortex clusters in S/F hybrids

**DOI:** 10.1038/srep38557

**Published:** 2016-12-09

**Authors:** C. Di Giorgio, F. Bobba, A. M. Cucolo, A. Scarfato, S. A. Moore, G. Karapetrov, D. D’Agostino, V. Novosad, V. Yefremenko, M. Iavarone

**Affiliations:** 1 “E.R. Caianiello” Physics Department, University of Salerno, Fisciano (SA), 84084, Italy; 2 Physics Department, Temple University, Philadelphia, PA 19122, United States; 3 CNR-SPIN Salerno, Fisciano (SA), 84084, Italy; 4 Physics Department, Drexel University, Philadelphia, PA 19104, United States; 5 Materials Science Division, Argonne National Laboratory, Argonne, IL 60439 United States; 6 National University of Science and Technology (MISiS), Moscow, 119049, Russia

## Abstract

While Abrikosov vortices repel each other and form a uniform vortex lattice in bulk type-II superconductors, strong confinement potential profoundly affects their spatial distribution eventually leading to vortex cluster formation. The confinement could be induced by the geometric boundaries in mesoscopic-size superconductors or by the spatial modulation of the magnetic field in superconductor/ferromagnet (S/F) hybrids. Here we study the vortex confinement in S/F thin film heterostructures and we observe that vortex clusters appear near magnetization inhomogeneities in the ferromagnet, called bifurcations. We use magnetic force microscopy to image magnetic bifurcations and superconducting vortices, while high resolution scanning tunneling microscopy is used to obtain detailed information of the local electronic density of states outside and inside the vortex cluster. We find an intervortex spacing at the bifurcation shorter than the one predicted for the same superconductor in a uniform magnetic field equal to the thermodynamical upper critical field H_c2_. This result is due to a local enhanced stray field and a competition between vortex-vortex repulsion and Lorentz force. Our findings suggest that special magnetic topologies could result in S/F hybrids that support superconductivity even when locally the vortex density exceeds the thermodynamic critical threshold value beyond which the superconductivity is destroyed.

Many of the potential applications of superconductors demand high current densities with minimal losses, a requirement that necessitates pinning of vortices. In the last decade several studies have focused on the influence of different types of pinning centers on the dynamics of superconducting vortices in type-II thin film superconductors^1^,^2^,^3^,^4^,^5^,^6^,^7^, which resulted in better fundamental understanding of vortex physics. Compared to nano-structured inclusions made of normal metals or insulators, magnetic pinning leads to intriguing effects, such as magnetostatic interaction^8^, proximity effect^9^ and domain wall superconductivity^10^,^11^,^12^,^13^, to name a few. Moreover, magnetic pinning in magnetically coupled superconductor/ferromagnet (S/F) heterostructures, appears to be stronger than other pinning mechanisms^4^,^14^. In particular, an enhanced pinning has been reported due to vortex coupling with ordered magnetic structures, such as stripes and dots^15^,^16^,^17^. Random point magnetic defects can act as strong local pinning centers as well. Topological defects and singularities are often present in the microscopic structure of magnetic materials as has been shown by a variety of imaging methods: optical microscopies (magnetic Kerr and Faraday), electron-based microscopies (Lorentz, spin resolved scattering and photoemission), X-ray microscopies (transmission and circular dichroism), local probe microscopies (magnetic force and spin polarized scanning tunneling microscopy)^18^,^19^,^20^,^21^,^22^,^23^,^24^,^25^,^26^. Some of these imaging techniques have been successfully used to investigate also the Abrikosov vortex distribution and vortex dynamics in superconducting thin films and S/F heterostructures^27^,^28^,^29^,^30^,^31^,^32^,^33^. Here, the existence of a stable mixed state results from the intrinsic repulsive interaction between superconducting vortices of the same polarity. It is known that in magnetic superconductors with easy magnetic axis, in anisotropic superconductors in tilted magnetic field and in high-κ S/F superlattices (where κ is the Ginsburg-Landau parameter, defined as the ratio of the London penetration depth *λ* to the coherence length *ξ*), attractive vortex-vortex interaction is responsible for the formation of vortex chains and vortex clusters^34^,^35^,^36^,^37^,^38^,^39^,^40^,^41^. Nevertheless, unconventional vortex configurations such as vortex chains, vortex clusters as well as multi-vortex and giant vortex phases can also be induced by a strong magnetic or geometric confinement potential^42^,^43^,^44^,^45^,^46^. The vortex confinement in peculiar superconductor/ferromagnet nanostructure formed by a superconducting microsquare with a magnetic disk on top has been theoretically addressed by Chen *et al*.^47^.

Up to now, low temperature magnetic force microscopy (MFM) has been a useful tool to image mesoscopic vortex configurations: vortex clusters pinned by a periodic array of magnetic dots in S/F structures^48^,^49^ as well as vortex-antivortex pairs and vortex chains in magnetically coupled planar S/F bilayers^50^,^51^,^52^,^53^. On the other hand, scanning tunneling microscopy and spectroscopy (STM/STS) has been successfully used by Karapetrov *et al*.^54^ to study vortex chain formation in S/F planar heterostructures and by Cren *et al*.^55^ to study the geometrical confinement effects on the stabilization of superdense multivortex and giant vortex phases in mesoscopic superconductors.

In this work we study the superconducting vortex confinement in planar S/F thin film heterostructures caused by intrinsic topological defects in the magnetic template called *bifurcations*, by low temperature magnetic force microscopy and scanning tunneling microscopy and spectroscopy. We demonstrate that a bifurcation can naturally lead to unusual vortex distribution, and eventually to the formation of vortex clusters, without any need of invasively engineering the shape of the sample via lithography or self-assembly. Moreover, we exploit the complementarities of low temperature MFM and STM/STS techniques to obtain a thorough understanding of the magnetic and electronic properties of these hybrid systems.

Indeed, MFM is sensitive to the changes in local magnetization in the superconductor on the length scale of London penetration depth λ, and it has proven to be a useful tool in studies of local magnetic behavior of ferromagnetic materials as well as imaging Abrikosov flux quanta distribution in superconductors or superconducting heterostructures. However, MFM has limited spatial resolution of few tenths of nanometers, strongly dependent on tip’s lift-height. On the other hand, STM/STS is sensitive to the amplitude of the superconducting order parameter, allowing detailed measurements of the electronic density of states (DOS) with a sub-nanometer spatial resolution, at the expense of not having any true magnetic vector contrast.

Here, we use MFM to get insight into the magnetic topology of a bifurcation as well as into its confinement power for superconducting vortices induced in the adjacent superconductor. At the same time we use STM/STS to get high resolution spatial maps of the electronic DOS of the superconductor just above the bifurcation sites in the ferromagnet.

We demonstrate that vortices above a bifurcation are closer than usual, producing vortex clusters and eventually reaching an intervortex distance which is even smaller than what expected at the higher critical field H_c2_. Such a result, never reported before in planar S/F heterostructures, is one of the main findings of the paper and can be explained as a consequence of the local enhancement of the magnetic out-of-plane stray field, occurring at the bifurcation, coupled to the peculiar bifurcation topology.

## Results

### Samples

Several magnetically coupled S/F heterostructures, made by Nb/Py and Pb/[Co/Pd]_n_ multilayers, were fabricated for MFM and STM/STS investigations. A thin insulating layer of 10 nm SiO_2_ or Al_2_O_3_ was used to electrically decouple the superconductor from the ferromagnet. This allows to study the effect of the spatially inhomogeneous stray field on the superconductivity without any interference from proximity coupling between the superconducting and magnetic layers^9^.

The thick Py films and Co/Pd multilayers are designed to have stripe magnetic domain pattern, with local magnetization vector alternating its projection along the out-of-plane direction between the adjacent magnetic stripes. In the Py, the in-plane easy magnetization axis is such that the canting angle of the magnetization vector is very small and magnetic stripe domains are formed only above the critical thickness of t_c_ ≈ 200–300 nm. Above this threshold, the magnetic stripe width *w* grows as square root of the film thickness *d*_*m*_, 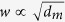
56,57. On the other hand, Co/Pd multilayers have a much stronger perpendicular anisotropy. The appearance of an out-of-plane stripe configuration of magnetic domains is a consequence of the competition between the perpendicular magnetic anisotropy and the thin-film shape anisotropy. Moreover, materials with low and medium perpendicular anisotropy, such as Py, always show twisted structure at the surface (*closure domains*) with an in-plane orientation of the magnetization, perpendicular to the domain wall, able to confine part of the out-of-plane magnetic flux and lower the magnetostatic energy^58^. The resulting domain structure consists of partially closed magnetic loops with alternating clockwise and counterclockwise orientation. In films with a strong perpendicular magnetic anisotropy the formation of closure domains becomes energetically unfavorable. However, recent magnetic simulations, based on the measured magnetic hysteresis loops, show the appearance of closure domains in a twin Co/Pd multilayers as well^59^. In general, closure domains are difficult to observe because the majority of the conventional magnetic imaging techniques rely on the detection of the out-of-plane stray fields. They could be detected by using Kerr microscopy technique, which is sensitive to the in-plane magnetic field component^60^.

It is worth noticing that the magnetic domain size and configuration in both ferromagnets are insensitive to the low magnetic fields applied during the field cooling processes performed in the MFM and STM/STS experiments here.

Twin superconducting films have been fabricated and characterized to derive the fundamental superconducting parameters. Twin Nb films have been magneto-electrically characterized, showing *ξ*_*Nb*_(0) ≈ 12 *nm* and *λ*_*Nb*_(0) ≈ 61 *nm*^50^,^53^. In such a case, Py films with thickness in the range of 1 ÷ 1.5 μm (and stripe widths w ≈ 500÷700 nm^53^) are good candidates for low temperature MFM experiments on Nb/Py hybrids. Indeed, being *λ*_*Nb*_(6K) ≈ 68 *nm*^53^ at the operational temperature of T = 6 K, vortices can be widely accommodated within the Py(1 ÷ 1.5 μm) stripe domains.

On the other hand, it is well known that bulk Pb exhibits type-I superconductivity (with *ξ*_*Pb*,bulk_(0) ≈ 83*nm* and *λ*_*Pb,bulk*_(0) ≈ 38 *nm*^61^). However, due to renormalization of the coherence length, Pb thin films behave as type-II superconductors and magnetic field penetrates in form of flux quanta. Moreover, in a twin 30-nm Pb film a ξ_*Pb*_(0) ≈ 48 *nm*^11^ has been measured using STM vortex imaging, in agreement with values of 40 ÷ 50 nm reported in literature for ultrathin Pb films^62^.

Different combination of superconductor and ferromagnet pairs were used in the S/F bilayers studied by MFM and STM/STS. For the superconducting materials, when limiting the choice to the simplest elemental superconductors, Nb and Pb are the best options. Indeed, due to their low intrinsic pinning, vortex arrangement can only be addressed to the ferromagnetic template. However, Nb is not a good candidate material for STM/STS investigation due to surface oxidation and difficulty to remove the oxide layer (possible only by annealing at temperatures higher than 1000 °C, which is not compatible with the underlying ferromagnet). On the other hand, Pb is not suitable for the MFM experiments, because of its low critical temperature that is too close to the minimum operational temperature of our instrument. Another important parameter to consider is the available scanning area. While STM provides the highest spatial resolution it does not allow large scanning areas since this compromises the stability of the tip-sample gap. Therefore, the largest STM scanning area of around 500 nm × 500 nm at T = 1.5 K requires a choice of ferromagnet with small stripe width and strong stray field, such as Co/Pd multilayers, to be able to visualize at least a few magnetic stripe domains within a single scan area and allow us to properly tune the intervortex distance with the applied magnetic field. However, Co/Pd multilayers are not suitable for MFM experiments, because of their relatively high out-of-plane stray magnetic fields inducing dense vortex arrangements that overlap on scale of λ.

In the following, MFM and STM/STS experiments on five S/F hybrids, Nb(100 nm, 150 nm, 200 nm)/Py(1 μm), Nb(150 nm)/Py(1.5 μm) and Pb(30 nm)/[Co(2 nm)/Pd(2 nm)]_200_, are being presented. The same samples have been used in our previous study of spontaneous vortex formation in the presence of regular magnetic template^53^,^63^. There we showed that vortices of opposite polarities form, i.e. vortices (V) and antivortices (AV), in absence of external magnetic fields whenever the out-of-plane magnetization of the ferromagnetic layer is above the threshold value calculated using the London approach to S/F hybrids in the mixed state^64^,^65^. In particular, MFM experiments proved that Nb(150 nm)/Py(1 μm), Nb(200 nm)/Py(1 μm) and Nb(150 nm)/Py(1.5 μm) have magnetization that is below the threshold value, preventing the formation of spontaneous V-AV pairs. In these samples Abrikosov vortex nucleation can be induced only by cooling in the presence of external magnetic field. On the other hand, in case of Nb(100 nm)/Py(1 μm) out-of-plane stripe magnetization exceeds the threshold value resulting in spontaneous vortex nucleation observed in low temperature MFM measurements^53^. Similarly, the presence of spontaneous V-AV structures in Pb(30 nm)/[Co(2 nm)/Pd(2 nm)]_200_ has been obtained in STM/STS experiments^63^. These samples are good candidates for studies of the confinement effects by magnetic bifurcations in cases of both spontaneous and external field-induced vortices.

### MFM observation of vortex clusters

**Figure 1:**
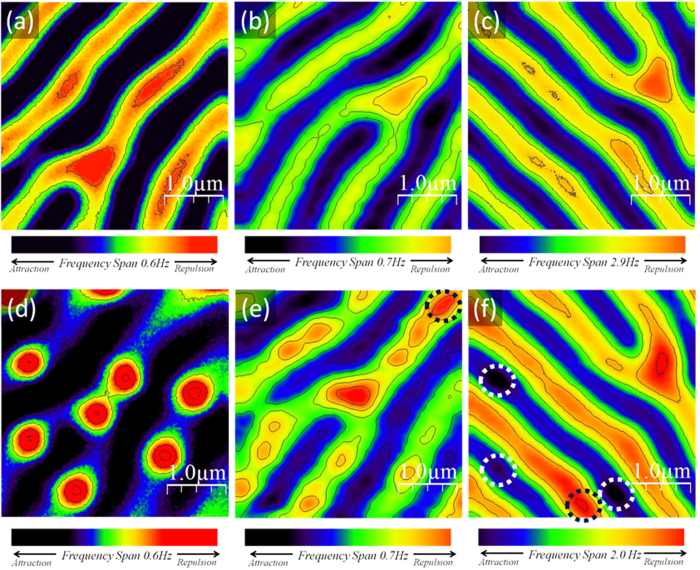
Vortex configurations at the bifurcation. (**a**)–(**b**)–(**c**) MFM maps acquired at T = 13 K and in zero applied magnetic field H = 0 on (**a**) Nb(150 nm)/Py(1 μm) at tip-sample distance h = 110 nm, (**b**) Nb(200 nm)/Py(1 μm) at h = 120 and (**c**) Nb(100 nm)/Py(1 μm) at h = 130. MFM maps acquired at T = 6 K and on (**d**) Nb(150 nm)/Py(1 μm) field cooled in the magnetic tip’s field, (**e**) Nb(200 nm)/Py(1 μm) field cooled in H = 30 Oe, (**f**) Nb(100 nm)/Py(1 μm) zero field cooled. Each map is 3.8 μm × 3.8 μm in size. In (**e**) the blue dotted circle points at a cluster of two vortices probably induced by a local Nb pinning. In (**f**), instead, blue and white dotted circles point at vortices and anti-vortices induced by the ferromagnet stray field.

Low temperature MFM measurements were performed on Nb/Py bilayers. Figure 1 shows the effect of the bifurcations on the vortex configurations in different situations. MFM maps acquired above and below the Nb critical temperature (T_s_ = 8.9K^53^) in samples with three different Nb layer thicknesses (150 nm, 200 nm, 100 nm) while keeping the Py layer 1 μm thick, are shown in Fig. 1. Above T_s_, at T = 13 K, the images show the stripe-like domain pattern of Py, with a stripe width w ≈ 500 nm, each of them containing dislocations of the regular magnetic structure. The significant increase of the magnetic contrast at the core of the bifurcation is representative of a stronger local stray field. Below the Nb T_s_, superconducting vortices are favored to nucleate at the bifurcation. Moreover, vortices always appear on a partially shielded magnetic stripe contrast. Indeed, the Nb thin films do not completely shield Py magnetic stray field, allowing the MFM visualization of the magnetic stripe domains even below T_s_. Nevertheless, a suppression of the magnetic contrast of the Py stripes due to the Nb diamagnetism, is confirmed by the comparison of the frequency span scale, above and below T_s_.

Figure 1(d) shows the MFM image acquired on Nb(150 nm)/Py(1 μm) at T = 6 K after a field cooling in the magnetic tip’s field, which is a non-uniform local dipole field (as shown in the Supplementary Fig. S1). This sample’s stripe magnetization is below the threshold for spontaneous formation of vortices and antivortices. Therefore, an external magnetic field is needed to facilitate nucleation of Abrikosov vortices. While cooling down below T_s_, the tip was kept in the bottom-left corner of the image, spaced by only a few nanometers from the sample surface. In such a case, vortices of the same magnetic polarity as the tip are induced within few microns around the tip’s apex. As shown in Fig. 1(d), a vortex is present in the middle of the dislocation, separated from its nearest neighbors by a distance sensibly smaller than the spacing between the vortices in the upper-right area of the image. Indeed, an average intervortex distance of about 1.2 μm is measured between flux quanta around the dislocation, whereas the intervortex spacing on the top of the bifurcation is reduced by 35% to about 780 nm. We infer that such effect is driven by the bifurcation topology and by the local enhancement of the Py stray field.

On the other hand, MFM maps acquired at T = 6 K in Nb(200 nm)/Py(1 μm) (Fig. 1(e)) and Nb(100 nm)/Py(1 μm) (Fig. 1(f)) show a strong magnetic contrast at the bifurcation site, surrounded by individual vortices nucleated in the presence of applied field of H = 30 Oe and in zero field cooling respectively. Line profile analysis performed on Nb(200 nm)/Py(1 μm) below T_s_ (Fig. 1(e)), confirms that superconducting vortices induced by the external field are mostly separated by a distance of (920 ± 105)nm, close to the expected value of 890 nm, derived by using 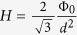
66, where *H* is the applied field, Φ_0_ is the flux quantum and *d* is the distance between vortices. Far from the dislocation area, a vortex cluster formed by two separate vortices, spaced by a distance of around 150 nm, appears in the map in Fig. 1(e) (outlined by a dark blue dotted circle). We infer that in this specific case the intrinsic pinning in the Nb layer causes a non-uniform vortex distribution.

On the other hand, spontaneous V/AV formation in Nb(100 nm)/Py(1 μm) is observed in Fig. 1(f), with vortices and antivortices induced by the stray fields of the Py stripes. The vortices are confined in blue and white dotted circles corresponding to vortex and antivortex confined to stripes with opposite magnetization polarity. Besides the vortices at the dislocation area, few spontaneous vortices populate the imaged area, being the out-of-plane magnetization of Py(1 μm) very close to the threshold value for spontaneous flux quanta nucleation in Nb(100 nm)/Py(1 μm)^53^. Careful analysis of the magnetic contrast at the dislocation is required to distinguish whether the observed magnetic profile represents a vortex clusters or a giant vortex.

**Figure 2:**
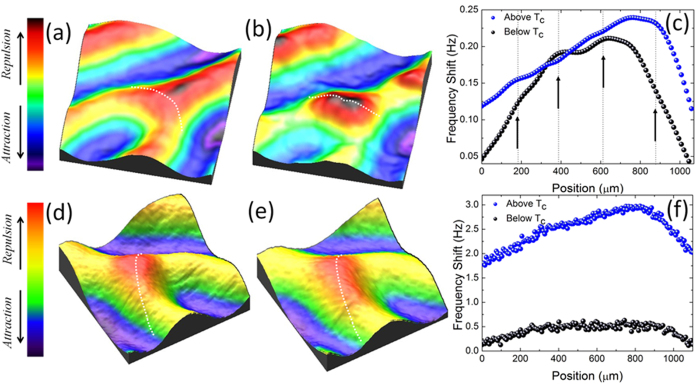
Magnetic contrast at magnetic stripe bifurcation. (**a)**–(**b**) 3D zoom-in of MFM maps acquired at T = 13 K and T = 6 K on Nb(200 nm)/Py(1 μm), focusing on the stripe bifurcation; (**c**) magnetic profile taken along white dotted lines of (**a**) and (**b**). Blue and black dots show the profiles above and below T_c_, respectively. Arrows point at possible vortex position inside the cluster. (**d**)–(**e**) 3D zoom-in of MFM maps acquired at T = 13 K and T = 6 K on Nb(100 nm)/Py(1 μm), focusing on the bifurcation; (**f**) magnetic profile taken along white dotted lines of (d) and (e). Blue and black dots draw the profiles above and below T_c_, respectively. Each map is 2 μm × 2 μm in size.

In Fig. 2, the 3D zoom-in of the MFM maps acquired on the top of the bifurcations in Nb(200 nm)/Py(1 μm) (Fig. 2 (a)–(b)) and Nb(100 nm)/Py(1 μm) (Fig. 2 (d)–(e)), above and below T_s_, are compared. Keeping the same tip-sample separation, the magnetic contrast of Nb(200 nm)/Py(1 μm) above and below the Nb superconducting transition appears quite different (Fig. 2(a,b)). A comparison of line profiles extracted from Fig. 2(a,b), above and below T_s_ is presented in Fig. 2(c). One observes significant change in the magnetic modulation. Indeed, two maxima of the magnetic roughness are clearly present below T_s_, while two more kinks can be observed closer to the borders (marked by arrows), which might suggests the presence of a more complex vortex cluster. The average intervortex spacing is about 200 nm, whereas the average distance far from the dislocation is about (920 ± 105) nm. Similar analysis performed on Nb(100 nm)/Py(1 μm), (Fig. 2(e)) does not show any drastic change in the magnetic roughness at the bifurcation core, compared to Fig. 2(d), acquired above T_s_ at the same tip-sample separation. The line profile comparison, reported on Fig. 2(f) and extracted from the white dotted profiles of Fig. 2(d)–(e), only confirms a reduction of the frequency shift below T_s_, due to Nb diamagnetism. However the formation of a superconducting vortex (giant or cluster) below T_s_ is determined from its interaction with the MFM tip when it moves closer to the Nb surface (Supplementary Fig. S2).

**Figure 3:**
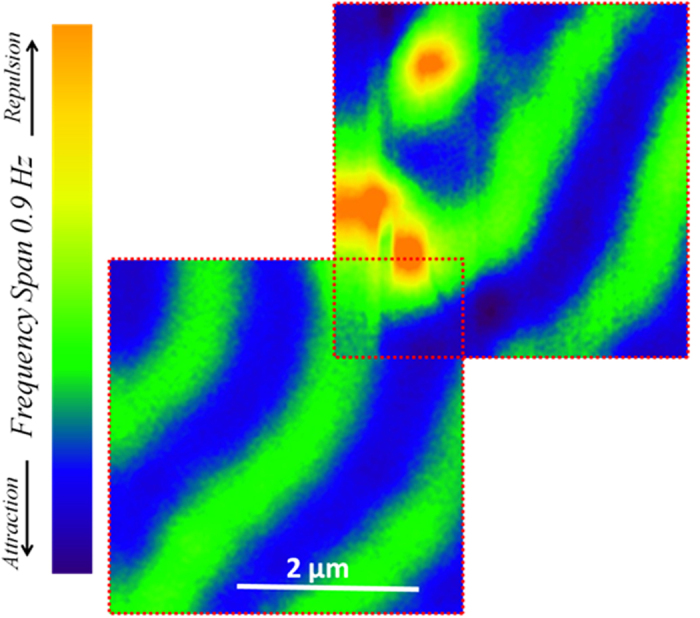
Local enhancement of stray field at the bifurcation. Patchwork of two MFM images acquired at T = 6 K and at a tip-sample distance of h = 180 nm, after a zero field cooling, in adjacent and partially overlapping locations on Nb(150 nm)/Py(1.5 μm) surface. Each map is 3.8 μm × 3.8 μm in size.

The MFM experiments performed on a S/F sample having a thicker Py layer, Nb(150 nm)/Py(1.5 μm), did not show any occurrence of spontaneous V-AV formation, proving that the equilibrium value of Py(1.5 μm) out-of-plane magnetization has to be lower than the threshold required to nucleate spontaneous vortices in Nb(150 nm)^53^,^64^,^65^. However, a strongly localized superconducting vortex nucleation can still be induced at the dislocation site, as shown in Fig. 3. Here, we present a patchwork made of two MFM maps of Nb(150 nm)/Py(1.5 μm) bilayer at T = 6 K after zero field cooling. In addition to the vortex nucleation on the top of the bifurcation, a vortex of opposite polarity (antivortex) is induced on the adjacent oppositely magnetized stripe and only a third red vortex appears in proximity of the dislocation. No other vortices (or antivortices) populate the imaged areas, confirming the magnetic influence of the dislocations on vortex nucleation. Indeed, not only that the vortex nucleation is favored at the bifurcation whenever the sample is over-threshold (Fig. 1(f)) or it is field cooled (Fig. 1(d)–(e)), but vortices could be locally induced by the dislocation even when no other spontaneous vortices are present. This also proves that the out-of-plane magnetization is under threshold everywhere except for the dislocation site, where a local enhancement of the stray field always occurs.

**Figure 4:**
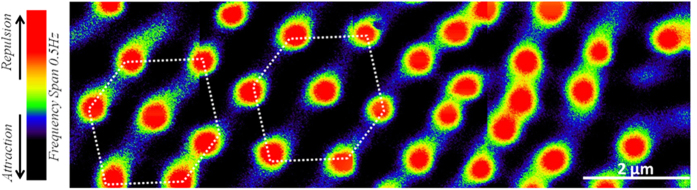
Topological influence of the dislocation on vortex configuration. Patchwork of four MFM images acquired at T = 6 K and h = 110 nm, after a field cooling in H = 19 Oe, in adjacent and partially overlapping locations on Nb(150 nm)/Py(1 μm) surface. The total size of the strip is 12 μm × 3.6 μm.

Finally, in Fig. 4, the topological influence of the bifurcation on vortex distribution is investigated. A strip of 12 μm × 3.6 μm in size, made by a patchwork of MFM images acquired at T = 6 K in four adjacent and partially overlapping locations on Nb(150 nm)/Py(1 μm) surface, is presented. The sample is field cooled in H = 19 Oe that corresponds to the first matching field 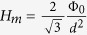
66 with intervortex distance *d* depending on the period of magnetization modulation in the ferromagnetic film (i.e. 
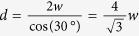
). Indeed, after the field cooling, vortices nucleate on the underlying magnetic domains of the same polarity as the external field. Wherever the magnetic stripe pattern is straight and regular, as in the left half-image of Fig. 4, the hexagonal arrangement of vortex lattice occurs with an average intervortex spacing of 1.2 μm, close to the expected value of 1.12 μm. In the right half-image the regularity of the hexagonal array is lost and an inhomogeneous distribution of flux quanta with a higher vortex density is observed. The intervortex spacing in this area is reduced by 40% to 700 nm. Such irregular vortex arrangement is driven by the presence of two dislocations of the magnetic stripe structure. The curvature of two stripes converging at the bifurcation acts as a vortex accumulation area increasing the local vortex density.

### STM/STS study of vortex clusters

Scanning tunneling microscopy and spectroscopy measures the local electronic density of states and could be used to discriminate between presence of Abrikosov vortex cluster and giant vortex. Despite the lack of magnetic field sensitivity, STM/STS allows vortex imaging with high lateral resolution up to very high magnetic fields. With STM one can infer the domain pattern in ferromagnetic layer in an indirect way: the nucleation sites of superconducting flux quanta after field-cooling the sample below T_s_ in opposite polarity magnetic fields, provides a unique fingerprint of the magnetic domains in the underlying ferromagnet^11^.

**Figure 5:**
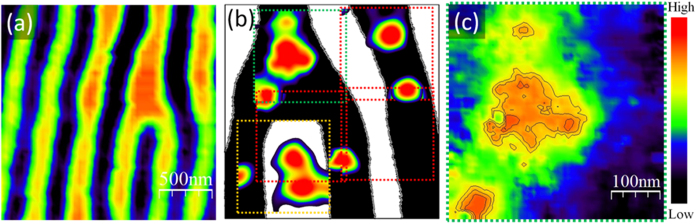
STM observation of vortex clusters at the bifurcation. (**a**) Room temperature MFM of [Co(2 nm)/Pd(2 nm)]_200_ multilayer; (**b**) spontaneous superconducting vortices in Pb(30 nm)/[Co(2 nm)/Pd(2 nm)]_200_ superimposed onto a cartoon of the magnetic domains. The vortices have been mapped by acquiring five conductance maps at the Fermi energy, each map being of 438 nm × 438 nm in size (T = 1.5 K after a zero field cooling); (**c**) conductance map at the dislocation (the location is the green dotted square in (**b**)), acquired at the Fermi energy and at T = 1.5 K in zero field cooling.

Low temperature STM/STS experiments were performed on Pb(30 nm)/[Co(2 nm)/Pd(2 nm)]_200_. As shown in Fig. 5(a) room temperature MFM on [Co(2 nm)/Pd(2 nm)]_200_ confirms a stripe-like configuration of magnetic domains with occasional dislocation defects. Figure 5(b) shows the position of spontaneous superconducting vortices in Pb(30 nm)/[Co(2 nm)/Pd(2 nm)]_200_ superimposed to a cartoon of the magnetic domains. This is a patchwork of five conductance maps of 438 nm × 438 nm in size, acquired at the Fermi energy at T = 1.5 K after zero field cooling. Vortices and antivortices appear as red spots with higher zero bias conductance (ZBC) compared to the superconducting background, which is here masked by the cartoon of the magnetic texture. An agglomeration of spontaneous flux quanta appears at the bifurcation core, where the vortex nucleation is favored because of the local stray field enhancement. The conductance map at the dislocation, in the green dotted square, at T = 1.5 K and in zero field cooling, is enlarged in Fig. 5(c). Three hot spots of conductance are present inside the cluster, which is surrounded by other two individual and isolated vortices. This strongly inhomogeneous vortex distribution is an evidence of the strong influence of magnetic defects on vortex arrangement.

**Figure 6:**
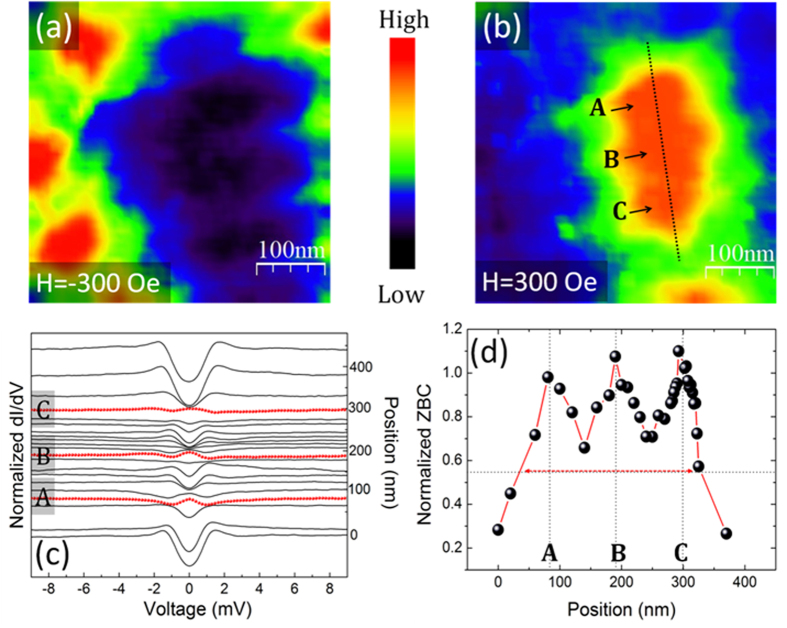
Vortex Cluster at the stripe endpoint revealed by STM. (**a**)–(**b**) Zero bias conductance maps acquired around the end point of an interrupted stripe at T = 1.5 K after field cooling in H = −300 Oe and H = 300 Oe, respectively; (**c**) plot of the spatial evolution of the electronic DOS along the dotted line in (**b**). Red dotted curves show pronounced zero bias peaks; (**d**) normalized ZBC vs position along the dotted line in (**b**).

In Fig. 6(a) we show ZBC maps acquired at T = 1.5 K in the yellow dotted area of Fig. 5(b) (just below the bifurcation) after a field cooling in H = −300 Oe and H = 300 Oe, respectively. A conductance distribution of individual and well-separated vortices appears in Fig. 6(a), while, in the opposite field antivortices agglomerate at the end point of the interrupted stripe (Fig. 6(b)). Here, spectroscopic analysis confirms the nucleation of a vortex cluster, formed by three individual flux quanta. The plot of Fig. 6(c) shows the evolution of the superconducting density of states (DOS) along the black dotted line of Fig. 6(b). The tunneling spectra gradually evolve while approaching the vortex cluster and they are characterized by an increase in the zero bias conductance and a decrease of the coherence peaks’ height. Clearly the superconductivity is fully suppressed in three separate locations, where a zero bias peak is observed (red dotted curves in Fig. 6(c)). The presence of a pronounced zero-bias peak in the conductance spectra at the vortex core is a clear evidence of the superconducting clean limit regime^67^,^68^. Finally, superconducting DOS features are completely recovered outside of the cluster. The plot of the normalized ZBC as a function of position, reported in Fig. 6(d), shows three ZBC peaks inside the cluster (indicated as A, B and C), separated by 105 nm (A and B) and 109 nm (B and C). Surprisingly, the resulting intervortex separation is about 1.4 times smaller than the minimum value possible, achievable at the second critical field *d(H*_c2_) ≈ 2.8 × ξ for fully separated vortices in bulk superconductors. Moreover, a rough estimate of *ξ*(1.5 *K*) can be deduced by the vortex profiles (red dotted arrow of Fig. 6(d)) resulting in *ξ*(1.5*K*) ≈ 50 *nm*, which is in good agreement with previous estimates on our twin samples^11^.

It is worth noticing that the size of the cluster that occupies the top of the bifurcation (Fig. 5(c)) is fully consistent with an agglomeration of three flux quanta, each of them having *ξ*(1.5*K*) ≈ 50 *nm* (Supplementary Fig. S3).

## Discussion

Direct observation of superconducting vortex cluster and vortex accumulation as a consequence of topological defects of the stripe magnetic structure in S/F thin film hybrids, made by Nb/Py and Pb/[Co/Pd]_n,_ has been carried out by using MFM and STM/STS techniques.

In general in S/F hybrids, with S in the mixed state, the position-dependent Lorentz force due to the screening currents above the magnetic domains, pushes the vortices to the center of the magnetic domains, thus minimizing the interaction energy between the superconducting vortices and the magnetic template^65^,^69^. In the presence of more than one flux quantum, the vortex-vortex repulsive interaction 
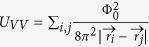
needs to be taken into account. In bulk superconductors vortices distribute in a hexagonal lattice, keeping a constant intervortex spacing given by 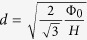. On the other hand in S/F hybrids with F in the stripe regime, the strong confinement exerted by the magnetic template leads to a chain-like distribution of vortices that eventually exhibit zig-zag displacement at high fields in wide stripes^50^. In such a scenario, vortex clusters can be explained as a consequence of several factors: a local stray field enhancement, a local reduction of the intervortex repulsive force as well as a local change in the Lorentz force action.

**Figure 7:**
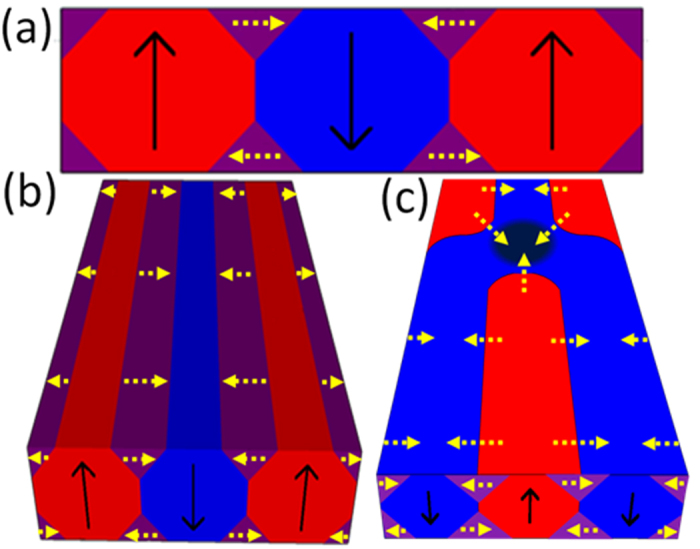
Schematics of the closure domains. (**a**)–(**b**) Front view and 3D schematics of an ideal magnetic stripe arrangement in the presence of closure domains; (**c**) schematics of the magnetic configuration around the bifurcation.

MFM measurements clearly show that a bifurcation, where two magnetic stripe domains converge and coalesce in a single one, leads to a local enhancement of the out-of-plane stray field. In Fig. 7(a) a front view and a 3D sketch of an ideal magnetic stripe arrangement is shown. Blue and red stripes are representative of magnetic domains with opposite out-of-plane polarization. Closure domains in the shape of triangular structures with an in-plane orientation of the magnetization (yellow dotted arrows) are at the sample surface depicted in purple. Such a magnetization distribution is consistent with the magnetic configuration of Co/Pd multilayers, whereas in Py an additional strong in-plane component of the magnetization along the stripes needs to be considered. Magnetic configuration around the bifurcation is presented in Fig. 7(c). The closure domains, indicated by the yellow dotted arrows, are forced to follow the domain wall curvature. As a consequence, an in-plane magnetic flux surplus (resulting from closure domains in Co/Pd multilayers and from closure domains and in-plane stripe magnetization in Py) needs to be expelled by flowing out from the sample surface via the out-of-plane stray field. This gives rise to the enhancement of the out-of-plane magnetization at the bifurcation core which leads to the enhancement of the local stray field that in-turn induces vortex clusters, as demonstrated by MFM and STM experiments.

Moreover, STS measurements on Pb/[Co/Pd]_n_ provided evidence for the existence of a vortex cluster spaced by a distance shorter than the minimum value achievable at the second critical field *d(H*_*c2*_) ≈ 2.8 × ξ. We infer that such a condition can be explained by taking into account the role of the bifurcation as topologically induced confinement. Indeed, while each vortex inside an infinite chain would feel the same net repulsive force, which leads to a constant intervortex distance, an unbalanced force is felt by vortices close to magnetic channel interruptions (stripe endpoint or bifurcation core). For instance, the vortex at the stripe endpoint feels a long-range repulsive interaction due to the semi-infinite vortex chain on one side, while on the other side only the Lorentz force would keep it away from the domain wall. In such a case a reduction of the intervortex distance close to the magnetic stripe endpoint is expected.

The bifurcation topology indirectly affects the vortex distribution at the nearest neighbor domains as well. As clearly shown in Fig. 4, hexagonal vortex lattice with an average lattice parameter of about 1.2 μm is achieved at the matching field^66^ wherever the stripes are straight and regular. On the other hand, around the dislocations the intervortex distance is affected by the stripe curvature, leading to a vortex-vortex spacing of about 700 nm. In the presence of straight magnetic domains, the Lorentz force vectors, being always normal to the domain walls where supercurrents flow, push vortices to the middle of the stripes. In such a case, the vortex distribution along the stripe is set by their repulsive interaction producing uniform intervortex spacing determined by the net magnetic field present - sum of the ferromagnet stray field and the external applied field. Instead, whenever there is a curvature of the domain walls, the Lorentz force vectors are locally not parallel, causing modulations of intervortex distance. At the bifurcation region the local bending of the stripes is extreme, leading to strong bending of the Lorentz force vectors that results in effective topological vortex confinement.

In conclusion, we have shown that randomly distributed defects in underlying magnetic structures, such as bifurcations, act as strong pinning sites that induce the nucleation of vortex clusters. Such dislocations strongly affect superconducting vortex distribution because of the magnetic confinement power coming from the local enhancement of the stray field and magnetic domain intrinsic topology. The topology of the magnetization at the bifurcation points in the ferromagnetic layer facilitates creation of vortex clusters in the superconductor, with vortices confined by the strong local Meissner currents while at the same time experiencing mutual repulsion.

## Methods

### Sample preparation

Nb/SiO_2_/Py were fabricated *ex-situ* by sputtering deposition and moved to the UHV chamber where the MFM experiments were performed. Py films were deposited by dc sputtering from a Ni_80_Fe_20_ target onto a Si substrate at a base pressure of 1.5 × 10^−7^ Torr, followed by a 10-nm SiO_2_ layer, in order to decouple the F from the S layer, suppressing proximity effects^9^. Nb films were deposited by dc sputtering at room temperature in a dedicated system with a base pressure of 2 × 10^−8^ Torr.

Samples for STM studies were fabricated in a different way to preserve their high surface quality. The [Co/Pd]_n_ multilayers (where *n* is the number of bilayers in the stack) were deposited *ex-situ* on Si substrates by dc sputtering in a dedicated system and in the presence of an applied in-plane magnetic field, favoring a stripe-like magnetic domain pattern. A 10-nm Al_2_O_3_ film was made by RF sputtering deposition from an Al target, in order to insulate the F from the S layer and suppress the proximity effect^9^. The 30-nm Pb film was deposited *in-situ* via e-beam evaporation at low temperature (120 K) and base pressure of 10^-11^ Torr, followed by a room temperature annealing. This procedure guaranties flat and clean surfaces, suitable for STM studies. The UHV chamber, where Pb films were made is linked to the STM chamber where the experiments were performed, in order to avoid surface contamination due to the exposure of the films to the air.

### Magnetic Force Microscopy

MFM experiments on Nb/Py where performed at T = 6 K using an Omicron cryogenic ultra-high vacuum (UHV) scanning force microscope, operating in frequency modulation-MFM mode. A commercial Si cantilever, equipped with a magnetic tip and having a resonance frequency f_0_ ≈ 75 kHz and elastic constant k ≈ 2.8 N/m, was used. The tip, coated by a ferromagnetic Co/Cr film, was characterized by nominal low moment μ ≈ 0.3 × 10^−13^emu and measured coercivity H_c,tip_ ≈ 550 ÷ 600 G. The magnetic imaging was done by scanning in non-contact regime and by mapping line by line the frequency shift df = f−f_0_ of the resonating cantilever, due to the stray field coming out from the sample (f is the oscillation frequency measured during tip-sample interaction and f_0_ is the force free resonant frequency of the cantilever). MFM maps were obtained by scanning at constant tip-sample heights, between 110 ÷ 180 nm, and the attractive/repulsive tip-sample interaction was mapped using color contrast. MFM spatial resolution is determined by the lift height used during the scanning and limited by the large magnetic volume of the tip.

### Scanning Tunneling Microscopy and Spectroscopy

STM/STS experiments were performed at T = 1.5 K by using a cryogenic UHV Unisoku USM-1300 system, equipped with RHK electronics. Tunneling spectroscopy was performed by using a standard lock-in technique with an alternating current modulation of 0.2 mV at 373 Hz. The conductance maps were acquired by scanning the tip over the sample surface at high voltage (20 mV), acquiring the lock-in signal at the Fermi energy in each location. Conductance maps reveal therefore, vortices by tracking the difference of the electronic density of states inside and outside the vortex cores. Topography was always acquired simultaneously to check the location where the spectroscopic information was recorded. All differential conductance spectra *dI/dV* were taken with the same tunneling parameters, with the junction stabilized at V = 10 mV, I = 100 pA.

## Additional Information

**How to cite this article**: Di Giorgio, C. *et al*. Observation of superconducting vortex clusters in S/F hybrids. *Sci. Rep.* **6**, 38557; doi: 10.1038/srep38557 (2016).

**Publisher's note:** Springer Nature remains neutral with regard to jurisdictional claims in published maps and institutional affiliations.

## Supplementary Material

Supplementary Information

## References

[b1] Otani Y., Pannetier B., Nozieres J. P. & Givord D. Magnetostatic interactions between magnetic arrays and superconducting thin films. J. Magn. Magn. Mater. 126, 622–625 (1993).

[b2] Baert M., Metlushko V. V., Jonckheere R., Moschalkov V. V. & Bruynseraede Y. Composite Flux-Line Lattices Stabilized in Superconducting Films by a Regular Array of Artificial Defects. Phys. Rev. Lett. 74, 3269–3272 (1995). 10058154 10.1103/PhysRevLett.74.3269

[b3] Martin J. I., Velez M., Hoffman A., Schuller I. K. & Vicent J. L. Artificially Induced Reconfiguration of the Vortex Lattice by Arrays of Magnetic Dots. Phys. Rev. Lett. 83, 1022–1025 (1999).

[b4] Milošević M. V. & Peeters F. M. Interaction between a superconducting vortex and an out-of-plane magnetized ferromagnetic disk: Influence of the magnet geometry. Phys. Rev. B 68, 094510-1–094510-12 (2003).

[b5] Karapetrov G., Fedor J., Iavarone M., Rosenmann V. & Kwok W. K. Direct Observation of Geometrical Phase Transitions in Mesoscopic Superconductors by Scanning Tunneling Microscopy. Phys. Rev. Lett. 95, 167002-1–167002-4 (2005). 16241833 10.1103/PhysRevLett.95.167002

[b6] Silhanek A. V. . Optimization of superconducting critical parameters by tuning the size and magnetization of arrays of magnetic dots. Phys. Rev. B 76, 100502-1–100502-4 (2007).

[b7] Gomez A. . Probing the dynamic response of antivortex, interstitial and trapped vortex lattices on magnetic periodic pinning potentials. Supercond. Sci. Technol. 26, 085018-1–085018-8 (2013)

[b8] Aladyshkin A. Y., Silhanek A. V., Gillijns W. & Moshchalkov V. V. Nucleation of superconductivity and vortex matter in superconductor–ferromagnet hybrids. Supercond. Sci. Technol. 22, 053001-1–053001-48 (2009).

[b9] Buzdin A. I. Proximity effects in superconductor-ferromagnet heterostructures. Rev. Mod. Phys. 77, 935–976 (2005).

[b10] Yang Z., Lange M., Volodin A., Szymczak R. & Moshchalkov V. V. Domain-wall superconductivity in superconductor–ferromagnet hybrids. Nat. Mater. 3, 793–798 (2004). 15467724 10.1038/nmat1222

[b11] Iavarone M. . Visualizing domain wall and reverse domain superconductivity. Nat. Comm. 5, 4766-1–4766-7 (2014). 10.1038/ncomms5766 PMC4354251 25164004

[b12] Aladyshkin A. Y. . Domain-wall superconductivity in hybrid superconductor-ferromagnet structures. Phys. Rev. B 68, 184508-1–184508-7(2003).

[b13] Werner R. . Domain-wall and reverse-domain superconducting states of Pb thin-film bridge on a ferromagnetic BaFe_12_O_19_ single crystal. Phys. Rev. B 84, 020505(R)-1–020505(R)-4 (2011).

[b14] Carneiro G. Pinning and creation of vortices in superconducting films by a magnetic dipole. Phys. Rev. B 69, 214504-1–214504-12 (2004).

[b15] Bulaevskii L. N., Chudnovsky E. M. & Maley M. P. Magnetic pinning in superconductor-ferromagnet multilayers. Appl. Phys. Lett. 76, 2594–2596 (2000).

[b16] Vlasko-Vlasov V. . Guiding superconducting vortices with magnetic domain walls. Phys. Rev. B 77, 134518-1–134518-7 (2008).

[b17] Vlasko-Vlasov V. . Domain structure and magnetic pinning in ferromagnetic/superconducting hybrids. Phys. Rev. B 85, 064505-1–0645015-15 (2012).

[b18] Fischery P. Imaging of magnetic domains by transmission x-ray microscopy. J. Phys. D: Appl. Phys. 31, 649–655 (1998).

[b19] Pietzsch O., Kubetzka A., Bode M. & Wiesendanger R. Observation of Magnetic Hysteresis at the Nanometer Scale by Spin-Polarized Scanning Tunneling Spectroscopy. Science 292, 2053–2056 (2001). 11408651 10.1126/science.1060513

[b20] Wachowiak A. . Direct Observation of Internal Spin Structure of Magnetic Vortex Cores. Science 298, 577–580 (2002). 12386329 10.1126/science.1075302

[b21] Murakami Y., Shindo D., Oikawa K., Kainuma R. & Ishida K. Magnetic domain structure in a ferromagnetic shape memory alloy Ni_51_Fe_22_Ga_27_ studied by electron holography and Lorentz microscopi. Appl. Phys. Lett. 82, 3695–3697 (2003).

[b22] Vansteenkiste A. . X-ray imaging of the dynamic magnetic vortex core deformation. Nature Physics 5, 332–334 (2009).

[b23] Kronast F. . Spin-resolved photoemission microscopy and magnetic imaging in applied magnetic fields. Surf. Interface Anal. 42, 1532–1536 (2010).

[b24] Jiang W. . Mapping the domain wall pinning profile by stochastic imaging reconstruction. Phys. Rev. B 87, 014427-1–014427-7 (2013).

[b25] Hierro-Rodriguez A. . Controlled nucleation of topological defects in the stripe domain patterns of lateral multilayers with perpendicular magnetic anisotropy. Phys. Rev. B 88, 174411-1–174411-9 (2013).

[b26] Blanco-Roldan C. . Nanoscale imaging of buried topological defects with quantitative X-ray magnetic microscopy. Nat. Comm. 6, 8196-1–8196-7 (2015). 10.1038/ncomms9196 PMC4569793 26337838

[b27] Volodin A. P. & Marchevsky M. V. Magnetic force microscopy investigation of superconductors: first results. Ultramiroscopy 42–44, 757–763 (1992).

[b28] Oral A., Bending S. J., Humphreys R. G. & Henini M. Vortex imaging in superconducting films by scanning hall probe microscopy. J. Low Temp. Phys. 105, 1135–1140 (1996).

[b29] Goa P. E. . Real-time magneto-optical imaging of vortices in superconducting NbSe_2_. Supercond. Sci. Technol. 14, 729–731 (2001).

[b30] Terao M., Tokunaga Y., Tokunaga M. & Tamegai T. Observation of single vortices by magneto-optical imaging. Physica C 426, 94–98 (2005).

[b31] Auslaender O. M. . Mechanics of individual isolated vortices in a cuprate superconductor. Nature Physics 5, 35–39 (2009).

[b32] Kramer R. B. G., Silhanek A. V., Gillijns W. & Moshchalkov V. V. Imaging the Statics and Dynamics of Superconducting Vortices and Antivortices Induced by Magnetic Microdisks. Phys. Rev. X 1, 021004-1–021004-7 (2011).

[b33] Brisbois J. . Imprinting superconducting vortex footsteps in a magnetic layer. Sci. Rep. 6, 27159 (2016). 27263660 10.1038/srep27159 PMC4893615

[b34] Auer J. & Ullmaier H. Magnetic Behavior of Type-II Superconductors with Small Ginzburg-Landau Parameters. Phys. Rev. B 7, 136–145 (1973).

[b35] Kogan V. G., Nakagawa N. & Thiemann S. L. Interaction of vortices in uniaxial superconductors. Phys. Rev. B 42, 2631–2634 (1990). 10.1103/physrevb.42.2631 9995736

[b36] Buzdin A. I. & Simonov A. Y. Magnetic flux penetration into layered superconductors. JETP Lett. 51, 1166–1171 (1990).

[b37] Buzdin A. I., Krotov S. S. & Kuptsov D. A. Attraction of inclined vortices in magnetic superconductors, Physica C:Supercond. 175, 1-2, 42–46 (1991).

[b38] Buzdin A. I. & Simonov A. Magnetization of anisotropic superconductors in the tilted magnetic field. Physica C 175, 143–155 (1991).

[b39] Bending S. J. & Dodgson M. J. W. Vortex chains in anisotropic superconductors. J. Phys.: Condens. Matter 17, R955–R993 (2005).

[b40] Bespalov A. A., Mel’nikov A. S. & Buzdin A. I. Clustering of vortex matter in superconductor-ferromagnet superlattices. EPL 110, 37003-p1–37003-p6 (2015).

[b41] Samokhvalov A. V., Savinov D. A., Mel’nikov A. S. & Buzdin A. I. Vortex clusters and multiquanta flux lattices in thin films of anisotropic superconductors. Phys. Rev. B 82, 104511-1–104511-15 (2010).

[b42] Tanaka K., Robel I. & Janko B. Electronic structure of multiquantum giant vortex states in mesoscopic superconducting disks. PNAS 998, 5233–5236 (2002). 16578872 10.1073/pnas.082096799 PMC122752

[b43] Lyuksyutov I. F. & Pokrovsky V. L. Ferromagnet–superconductor hybrids. Advances in Physics 541, 67–136 (2005).

[b44] Zhang L. F., Covaci L., Milosevic M. V., Berdiyorov G. R. & Peeters F. M. Vortex states in nanoscale superconducting squares: The influence of quantum confinement. Phys. Rev. B 88, 144501-1–144501-13 (2013).

[b45] Cieplak M. Z. . Tuning vortex confinement by magnetic domains in a superconductor/ferromagnet bilayer. Phys. Rev. B 87, 014519-1–014519-13 (2013).

[b46] Cren T., Fokin D., Debontridder F., Dubost V. & Roditchev D. Ultimate Vortex Confinement Studied by Scanning Tunneling Spectroscopy. Phys. Rev. Lett. 102, 127005-1–127005-4 (2009). 19392315 10.1103/PhysRevLett.102.127005

[b47] Chen Q. H., Carballeira C. & Moshchalkov V. V. Vortex matter in a hybrid superconducting/ferromagnetic nanostructure. Phys. Rev B 79, 104520-1–104520-12 (2009).

[b48] Shapoval T. . Enhanced pinning of superconducting vortices at circular magnetic dots in the magnetic-vortex state. Physica C 470, 867–870 (2010).

[b49] Shapoval T. . Direct observation of superconducting vortex clusters pinned by a periodic array of magnetic dots in ferromagnetic/superconducting hybrid structures. Phys. Rev. B 81, 092505-1–092505-4 (2010).

[b50] Iavarone M. . Imaging the spontaneous formation of vortex-antivortex pairs in planar superconductor/ferromagnet hybrid structures. Phys. Rev B 84, 024506-1–024506-5 (2011).

[b51] Iavarone M. . Vortex Confinement in Planar Superconductor/Ferromagnet Hybrid Structures. IEEE Trans. Magn. 48, 3275–3279 (2012).

[b52] Cucolo A. M. . Visualizing Vortex Dynamics in Py/Nb Thin Film Hybrids by Low Temperature Magnetic Force Microscopy. J. Supercond. Nov. Magn. 25, 2167–2171 (2012).

[b53] Bobba F. . Vortex-antivortex coexistence in Nb-based superconductor/ferromagnet heterostructures. Phys. Rev. B 89, 214502-1–214502-7 (2014).

[b54] Karapetrov G., Milosevic M. V., Iavarone M., Fedor J., Belkin A., Novosad V. & Peeters F. M. Transverse instabilities of multiple vortex chains in superconductor-ferromagnet bilayers. Phys. Rev. B 80, 180506-1–180506-4 (2009).

[b55] Cren T., Serrier-Garcia L., Debontridder F. & Roditchev D. Vortex Fusion and Giant Vortex States in Confined Superconducting Condensates. Phys. Rev. Lett. 107, 097202-1–097202-5 (2011). 21929264 10.1103/PhysRevLett.107.097202

[b56] Murayama Y. Micromagnetics on Stripe Domain Films. I. Critical Cases. J. Phys. Soc. Jap. 21, 2253–2266 (1966).

[b57] Chikazumi S. Physics of Ferromagnetism, Oxford University Press Inc., New York (1997).

[b58] Kittel C. Physical theory of ferromagnetic domains. Rev. Mod. Phys. 21, 541–583 (1949).

[b59] Moore S. A. . Doppler-scanning tunneling microscopy current imaging in superconductor-ferromagnet hybrids. Appl. Phys. Lett. 108, 042601-1–042601-5 (2016).

[b60] Hubert A. & Schäfer R. Magnetic Domains, Springer (1998).

[b61] Klein O., Nicol E. J., Holczer K. & Gru¨ner G. Conductivity coherence factors in the conventional superconductors Nb and Pb. Phys. Rev. B 50, 6307–6316 (1994). 10.1103/physrevb.50.6307 9977008

[b62] Zhang T. . Superconductivity in one-atomic layer metal films grown on Si(111). Nat. Phys. 6, 104–108 (2010).

[b63] Iavarone M., Moore S. A., Fedor J., Novosad V., Pearson J. A. & Karapetrov G. Influence of Domain Width on Vortex Nucleation in Superconductor/Ferromagnet Hybrid Structures, J. Supercond. Nov. Magn. 28, 1107–1110 (2015).

[b64] Genkin G. M., Skuzovaktin V. V. & Tokman I. D. Magnetization of the ferromagnetic-superconductor structures. J. Magn. Magn. Mater. 130, 51–56 (1993).

[b65] Laiho R., Lähderanta E., Sonin E. B. & Traito K. B. Penetration of vortices into the ferromagnet/type-II superconductor bilayer. Phys. Rev. B 67, 144522-1–144522-7 (2003).

[b66] Tinkham M. Introduction to Superconductivity, 2^nd^ edtion, McGrow-Hill, Inc. (1996).

[b67] Hess H. F., Robinson R. B., Dynes R. C., Valles J. M. Jr. & Waszczak J. V. Scanning-Tunneling-Microscope Observation of the Abrikosov Flux I.attice and the Density of States near and inside a Fluxoid. Phys. Rev. Lett. 62, 214–216 (1989). 10039952 10.1103/PhysRevLett.62.214

[b68] Shore J. D., Huang M., Dorsey A. T. & Sethna J. P. Density of States in a Vortex Core and the Zero-Bias Tunneling Peak. Phys. Rev. Lett. 6226, 3089–3092 (1989). 10040175 10.1103/PhysRevLett.62.3089

[b69] Sonin E. B. Comment on “Ferromagnetic film on a superconducting substrate”. Phys. Rev. B 66, 136501-1–136501-3 (2002).

