# Supplementary material for: Observation of superconducting vortex clusters in S/F hybrids
Source: Sci Rep. 2016 Dec 9;6:38557. doi: 10.1038/srep38557 (PMC5146919; doi:10.1038/srep38557)
Supplement: Supplementary Information [file srep38557-s1.pdf]

Observation of superconducting vortex clusters in S/F hybrids

C. Di Giorgio[1,2*], F. Bobba[1,3] , A.M. Cucolo[1,3], A. Scarfato[1,†], S.A. Moore[2], G. Karapetrov[4], D. D'Agostino[1], V. Novosad[5], V. Yefremenko[5] and M. Iavarone[2]

[1.] "E.R. Caianiello" Physics Department, University of Salerno, Fisciano (SA), 84084, Italy

[2.] Physics Department, Temple University, Philadelphia (PA), 19122, United States

[3.] CNR-SPIN Salerno, Fisciano (SA), 84084,Italy

[4.] Physics Department, Drexel University, Philadelphia (PA), 19104, United States

[5.] Materials Science Division, Argonne National Laboratory, Argonne (IL), 60439 United States

*cdigiorgio@unisa.it

Supplementary Information

Magnetic tip's field

Supplementary Fig. S1 shows a patchwork of MFM maps acquired in three adjacent and partially overlapping regions of Nb(150nm)/Py(1μm) at T=6K. The sample was simultaneously field cooled in the tip's field and in an opposite external field of 11.5Oe. During the cooling down, the tip apex was kept at the left-bottom corner of the scan size. Below the Nb critical temperature T_s, no superconducting vortices are observed close to the initial tip position. The absense of both V (concord to tip's field) and AV (concord to the external magnetic field) indicates that the two magnetic fields are locally compensating each other. By moving away from the bottom-left corner an AV, with the same polarity of the external field, appears, being the proof that the tip's magnetic field is slowly decreasing. The AV density gradually increases moving far from the initial tip position.

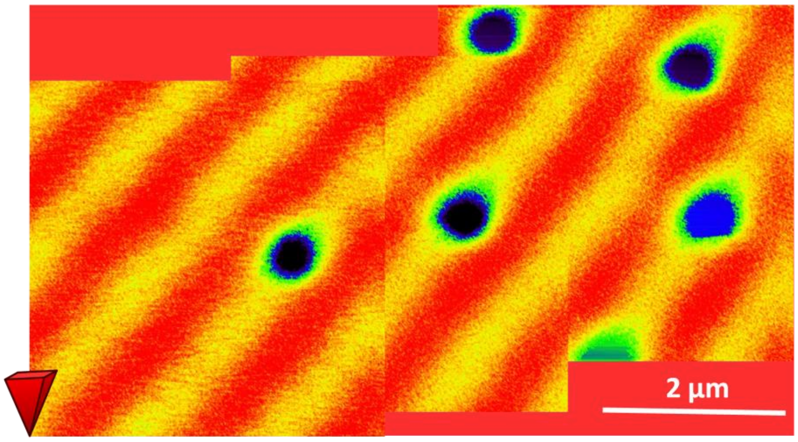

Supplementary Figure S1. Vortices induced by the tip's magnetic field. Patchwork of three MFM images acquired on Nb(150nm)/Py(1.5µm) at T=6K, after a simultaneous field cooling in tip's field and opposite external field of 11.5Oe. Each map is 3.8µm ×3.8µm in size. Tip position during the cooling down is indicated by the reversed pyramid in bottom-left corner.

Vortex motion

Supplementary Fig. S2 shows a comparison of MFM maps acquired on Nb(100nm)/Py(1µm) at T=6K and at tip-sample separation of 130nm (Fig. S2(a)) and 30nm respectively (Fig. S2(b)). In general, by moving the tip closer to the Nb surface, its magnetic interaction with the vortex is favored. Sometimes this interaction can be strong enough to cause vortex motion. Such events are imaged as instabilities in the MFM map. This technique was used during the imaging of the dislocation region, in order to determine if the magnetic contrast observed was due to a superconducting vortex (giant or cluster) nucleated at the bifurcation core. By scanning with a tip-sample separation of 30nm a jump is recorded in the MFM image, which can only be addressed to vortex motion. Indeed such features are never detected at higher scanning height or above the Nb superconducting critical temperature.

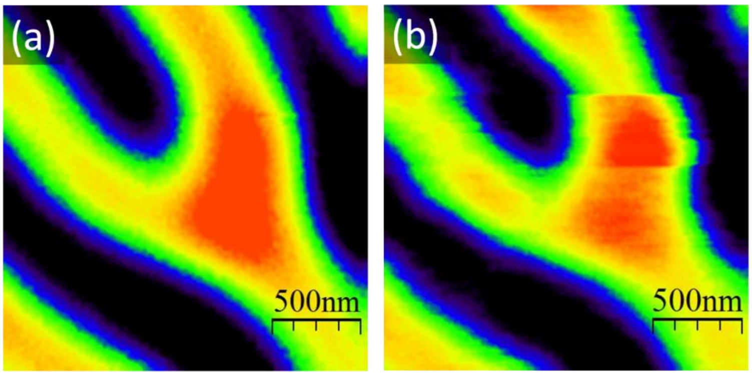

Supplementary Figure S2. Vortex motion. MFM maps of Nb(100nm)/Py(1µm) at T=6K, H=0 Oe and tip-sample separation of (a) 130nm and (b) 30nm. Each map is 2µm ×2µm in size.

STM Conductance map.

Supplementary Fig. S3(a) shows an STM conductance map of Pb(30nm)/ [Co(2nm)/Pd(2nm)]$_{200\text{-bilayers}}$ at the bifurcation core of the stripe-like magnetic pattern underneath. It was acquired at the Fermi energy, at T=1.5K, after a zero field cooling and it shows a vortex agglomeration due to the local stray field enhancement. In Fig. S3(b) raw data have been filtered by a two-standard deviation 2D Gaussian smoothing, with a kernel size of 15 pixels, corresponding to 105nm, and reported in a black-white color scale, with a completely satured contrast of the superconducting background. Red circles, with diamater fixed by 2ξ (with $\xi(1.5K) \approx 50nm$), on the top of the map, work as guide for the eye in order to check the number of vortices inside the agglomeration. Coerently with the three conductance "hot spots" of Fig. S3(a), the cluster size is recovered only when the confinament of three flux quanta is considered.

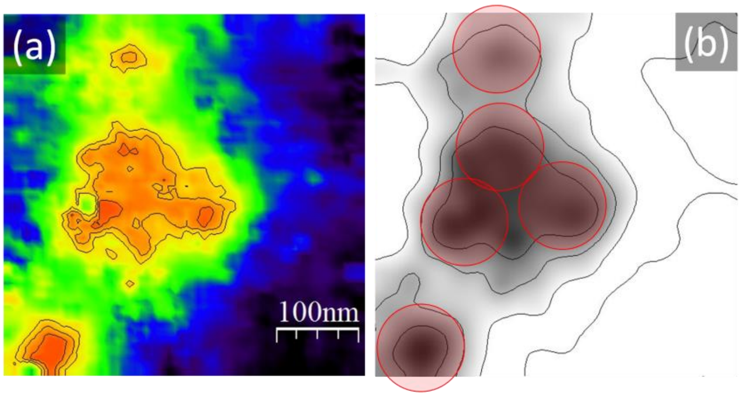

Supplementary Figure S3. STM observation of a vortex cluster (a) Conductance map at the bifurcation core, acquired at the Fermi energy at T=1.5 K after a zero field cooling. (b) Gaussian smoothing of the raw data. Kernel size =15 pixel, 105nm. Numebr of standard deviations=2. Red cicles outline the position of superconducting vortices. Maps size: 438nm×438nm.
